# Stigmatization profiles and psychological distress in people at high risk of infection with COVID-19 –A study conducted in Germany from March to August 2021

**DOI:** 10.1371/journal.pone.0285788

**Published:** 2023-05-18

**Authors:** Sandrine Bisenius, Julia Treml, Franz Hanschmidt, Anette Kersting

**Affiliations:** Department of Psychosomatic Medicine and Psychotherapy, University of Leipzig, Leipzig, Saxony, Germany; Sapienza University of Rome, ITALY

## Abstract

COVID-19-related stigmatization of affected people or people at risk of infection has been shown to enhance the reluctance of affected individuals to use health services and reduce their mental health. It is thus highly important to gain a thorough understanding of COVID-19-related stigmatization. The present study’s first aim was to explore stigmatization profiles of experienced stigmatization (anticipated stigmatization, internalized stigmatization, enacted stigmatization, disclosure concerns) and stigmatization practices in 371 German people at high risk of infection using latent class analyses. The second aim was to investigate the relationship between stigmatization profiles and psychological distress via multiple regression analysis taking into account other possible negative and positive risk factors. Our results showed two stigmatization profiles: "high stigmatization group" and "low stigmatization group". Belonging to the "high stigmatization group" was significantly correlated with higher levels of psychological distress. Other risk factors significantly related to psychological distress were mental health disorders in the past, exposure to COVID-19, fear related to COVID-19, perceived risk of being infected, lower perceived self-efficacy, and lower subjective knowledge about COVID-19.

## 1. Introduction

Since the declaration of the pandemic by the WHO in March 2020, COVID-19, caused by the severe acute respiratory syndrome coronavirus 2 (SARS-CoV-2), has been shown to spread rapidly and claimed over 6 million deaths worldwide [1]. In order to contain the pandemic, governments worldwide imposed lockdowns, quarantine, isolation, and public health measures [2–4]. Although effective in preventing the further spread of SARS-CoV-2, these measures largely changed behavioral, social, and economic aspects of people’s everyday lives [2,5]. These profound changes in everyday life, together with the health threat posed by COVID-19, have been related to increased mental health problems like stress disorders, sleep disorders, psychological distress, depressive symptoms and anxiety symptoms [2–7].

MentaI health problems during the COVID-19 pandemic, especially psychological distress, depressive symptoms, and anxiety symptoms, have been associated with risk factors like female gender, younger age, lower education, comorbid physical health condition, history of psychological distress or mental health disorder, fear or worries related to COVID-19, lower perceived personal self-efficacy, risk perception, knowledge about COVID-19 and COVID-19-related stigmatization [6,8–10].

Stigmatization has been defined as the process of devaluing individuals who possess "an attribute that is deeply discrediting" either because it is considered to transgress the norms of society or to endanger the safety or health of other people [11–13]. According to the Health Stigma and Discrimination Framework, two kinds of stigmatization can be differentiated: stigmatization experiences and stigmatization practices [14]. Stigmatization experiences include experienced stigmatization and discrimination through other people, internalized stigmatization, perceived stigmatization (perceptions about how stigmatized groups are treated), anticipated stigmatization, and secondary stigmatization (experience of stigmatization by family or friends), while stigmatization practices include stereotypes, prejudice, discriminatory attitudes, and stigmatization behavior. In cases where the attribute considered "discreditable" is not immediately apparent to others, the individual has the opportunity to conceal it in order to avoid stigmatization [13,15]. Although the resulting "disclosure concerns" have not been explicitly mentioned within the Health Stigma and Discrimination Framework, we consider them a further facet of stigmatization experiences.

COVID-19-related stigmatization and discrimination affect patients/infected people, recovered patients, and people at high risk of infection (e.g., healthcare workers, frontline government workers, family members of COVID-19 patients, or people returning from a high-risk area) [16–21]. The reported stigmatization and discrimination experiences range from internalized stigmatization, verbal discrimination, workplace discrimination, denied access to public transport, or termination of the rented apartment to physical harm.

According to a recent meta-analysis, most studies investigating stigmatization have been conducted in low- and middle-income countries, and there is still a lack of knowledge about stigmatization related to COVID-19 in high-income countries [6]. Furthermore, little is known about the quantitative estimates of pandemic-related stigmatization profiles.

One possibility to provide stigmatization profiles is using latent class analysis (LCA), a data-driven, person-centered approach that reveals underlying subgroups within an overall sample [22]. The individuals within a subgroup are similar to each other regarding the variables entered in the analysis and different from the individuals in the other subgroups. So far, there is only one study using LCA to characterize stigmatization profiles within the context of COVID-19 [23].

The first aim of our exploratory study was to characterize the stigmatization profiles of anticipated stigmatization, internalized stigmatization, enacted stigmatization, disclosure concerns, and stigmatization practices in a German study sample of people at high risk of infection with COVID-19 using latent class analysis. High risk of infection was defined as a) contact with an infected person, b) symptoms attributable to COVID-19, or c) returning from traveling in a high-risk area. To our knowledge, this is the first study providing a detailed profile of COVID-19-related stigmatization and one of the first studies using LCA in this context. The second aim of our study was to investigate the relationship between stigmatization profiles and psychological distress while taking into account other positive and negative risk factors possibly related to psychological distress.

## 2. Materials and methods

### 2.1 Recruitment of the study sample

This study is part of an online survey investigating the effects of stigmatization related to COVID-19 on psychological distress and on adherence to behavioral recommendations by the government in a German population at high risk of being infected with COVID-19.

Recruitment was carried out by convenience sampling from 03/2021 to 08/2021 via advertisements for participation in this study (including the link to the survey) posted in Facebook groups (general survey groups and general bulletin boards) and on COVID-19 websites of German cities and municipalities. During the last six weeks of the study period, the same advertisements were additionally posted on websites of companies and leisure clubs in Leipzig. During the time of the study, the German government implemented restrictions on the number of people allowed to meet in public, introduced a safety distance of 1.5–2.0 m between people in public spaces, and gave recommendations on how to avoid contagion. To be eligible for participation, subjects had to 1) be older than 18 years, 2) have a place of residence in Germany, 3) be fluent in speaking German, and 3) have been at risk of being infected with SARS-CoV-2 after 11/2020 by either a) having had contact with a person tested positively for COVID-19, b) having suffered from symptoms that might be due to COVID-19 (i.e., cough, increased temperature or fever, dyspnea, loss of smell and taste, cold, sore throat, headache, muscle pain, joint pain, weakness/fatigue) or c) entering Germany after returning from traveling in a high-risk area for COVID-19. As an incentive, participants could participate in a lottery for ten book tokens (€10). The study included electronic informed consent in alignment with the Declaration of Helsinki, was registered in the German Clinical Trial Register (DRKS00029552), and was approved by the University of Leipzig Ethics Committee (032/21-ek; 16.02.2021).

### 2.2 Sociodemographic and health-related data

Sociodemographic data included age, gender, nationality, educational level (less or more than 12 years), employment status (yes/no), partnership status (yes/no), and the number of people living in the household. Participants were furthermore asked about mental health disorder(s) in the past (yes/no), chronic illness (yes/no), date of the critical event (of possibly having been infected with COVID-19), and whether they had been tested positively for COVID-19 after the critical event.

### 2.3 Stigmatization

As at the time of recruitment no standardized stigmatization scales for COVID-19 were available, stigmatization items were adapted from validated stigma scales for HIV and chronic illness [24–27]. Participants were asked to remember the 24 h after having been at risk of being infected with COVID-19 and rate how much they agreed with the respective statement.

Anticipated stigma was assessed via six items: "I worried that a friend or family member might become angry at me if I had COVID-19 "[1 = totally disagree-4 = totally agree], "I worried that a friend or family member would blame me for having COVID-19 "[1 = totally disagree-4 = totally agree], "I worried that a friend or family member would think it was my fault that I got COVID-19 "[1 = totally disagree-4 = totally agree], "I worried that I would be discriminated against at work/university/educational training if I had COVID-19 "[1 = totally disagree-4 = totally agree, 5 = not applicable], "I worried that someone at work/university/educational training would blame me for having COVID-19 "[1 = totally disagree-4 = totally agree, 5 = not applicable], "I worried that my superior would fire me "[1 = totally disagree-4 = totally agree, 5 = not applicable]. Cronbach’s alpha for the anticipated stigmatization scale was 0.83.

The internalized stigmatization scale consisted of 7 items rated on a 4-point Likert scale [1 = totally disagree-4 = totally agree]: "I felt like a bad person ", „I felt like I’m not as good as others“,“I thought badly about myself“, „I felt guilty“, „I felt ashamed“, „I felt unclean“, „The thought of an infection with COVID-19 disgusted me“. Cronbach’s alpha for the internalized stigmatization scale was 0.90.

Disclosure concerns were measured via three items on a 4-point Likert scale [1 = totally disagree-4 = totally agree]: „I was very careful whom I told about the critical event, “I felt like I needed to hide the fact that the critical event took place”, „I worried that people who knew that the critical event took place would tell others“, with critical event referring to a) having had contact with a person tested positively on COVID-19, b) having suffered from symptoms that might be due to COVID-19, or c) entering Germany after returning from traveling in a high-risk area for COVID-19. Cronbach’s alpha for the disclosure concerns scale was 0.86.

Enacted stigmatization was assessed with two items: „I felt hurt by how people reacted after knowing that the critical event took place” [1 = totally disagree-4 = totally agree, 5 = not applicable], „I regret having told certain people that the critical event took place” [1 = totally disagree-4 = totally agree, 5 = not applicable], with the critical event being defined as above. Cronbach’s alpha and Spearman-Brown’s coefficient for enacted stigmatization were 0.81.

The stigmatization practices scale included four items rated on a 4-point Likert scale [1 = totally disagree-4 = totally agree]: „People infected with COVID-19 should feel ashamed of themselves“, „People infected with COVID-19 must have done something wrong to deserve it“, „People infected with COVID-19 have themselves to blame“, „A COVID-19 infection is a punishment for bad behavior“. Cronbach’s alpha for the stigmatization practices scale was 0.77.

### 2.4 Psychological variables

Fear related to COVID-19, perceived self-efficacy, perceived risk of being infected with COVID-19, and subjective knowledge about COVID-19 were assessed by the following single items: “During the first 24 hours after the critical event, I perceived the coronavirus as. . . [1 = not frightening-7 = frightening], “During the first 24 hours after the critical event, I considered the coronavirus as something that …[1 = made me feel helpless-7 = I could actively cope with“], “During the first 24 hours after the critical event, how likely did you consider the risk of infection with COVID-19 [1 = very unlikely-7 very likely]?”, “During the first 24 hours after the critical event, how much knowledge about COVID-19 did you have?”[1 = no knowledge at all-7 = a large amount of knowledge“].

Psychological distress was measured with the Patient Health Questionnaire-4 [28,29]. The PHQ-4 consists of the 2-item Patient Health Questionnaire (PHQ-2) assessing the two core criteria for depressive disorders and the 2-item Generalized Anxiety Disorder Screener (GAD-2) tapping into the two core criteria for generalized anxiety disorder. Cronbach’s alpha of the total score in our study sample was 0.86.

### 2.5 Statistical analyses

Missing data inspection, inspection of distributions, internal consistency analyses of the stigmatization scales, and descriptive statistical analyses were performed using SPSS Version 27 [30].

Latent class models were computed with Mplus 7.4 [31]. Due to sparse data, the means of the single stigmatization scales were dichotomized into less than 2.5 (disagree) and more than 2.5 (agree) before entering the analyses. After checking for non-violation of the local independence assumption, we estimated latent class models via robust maximum likelihood estimation, choosing 3000 random starts. The estimation process started with two latent classes and was increased to three classes while comparing model fits between models. We chose 50 random starts with 50 bootstrap draws for the bootstrapped likelihood ratio test (BLRT), comparing the estimated model to a model with one less class. The results of the latent class analyses were evaluated based on Akaike’s information criterion (AIC), Bayesian Information Criterion (BIC), Vuong-Lo-Mendell-Rubin adjusted likelihood ratio (VLMR), BLRT, average latent class probabilities, entropy value, and theoretical interpretability [22,32–34]. Lower AIC and BIC values indicate a better resulting statistical model, while significant VLMR and BLRT values indicate that the current model fits significantly better than a model with one less class [33,34]. Average latent class probabilities and entropy values indicate how well participants have been classified, with an entropy higher than 0.6 being considered good [22,32]. All analyses were based on alpha = 0.05. Subsequently, a binary logistic regression with stigmatization class as the dependent variable and demographic variables and psychological variables as independent variables was conducted, as well as a linear regression analysis with PHQ-4 sum score as the dependent variable and demographic and psychological variables (including the stigmatization classes) as independent variables. For analysis purposes, categorical demographic variables were dummy-coded. All regression analyses were based on alpha = 0.05 and conducted in SPSS Version 27 [30].

## 3. Results

### 3.1 Study sample

The conservatively calculated minimum sample size required to examine the selected variables in the adult German population (69,434,451 inhabitants older than 18 years) consisted of 385 participants assuming a response distribution of 50%, a 95% confidence level, and a 5% margin error [35]. Of the 429 subjects who gave electronic informed consent and fulfilled the inclusion criteria, 57 (13.29%) participants were excluded due to missings on core variables, with “not applicable” being considered as missings. Subsequently, the gender category nonbinary was excluded from further analyses as only one participant endorsed this category. Thus, the final study sample consisted of 371 subjects, slightly below the targeted minimum sample size of 385 participants. Of our study sample, most participants were female (79.8%), well-educated people with more than 12 years of education (74.4%), people that were employed (84.9%), and people who were in a partnership (76.8%). Approximately 16% of the participants reported anticipated stigmatization, 11% disclosed internalized stigmatization, 31% reported disclosure concerns, 20% stated having experienced enacted stigmatization, and 3.5% reported stigma practices. Levels of psychological distress in our study sample were moderate (PHQ-4 sum score: 8.42 ± 3.47; scale range: 0–12). Descriptive statistics for the study sample are shown in Table 1.

**Table 1:** Characteristics of the overall study sample.

	overall sample (n = 371)
	mean (± SD) / n (%)
**age** (range 18–73 years)	42.40 (11.69)
**Gender**	
Male	75 (20.2)
Female	296 (79.8)
**Nationality**	
German	369 (99.5)
Other	2 (0.5)
**education level**	
< 12 years	95 (25.6)
> 12 years	276 (74.4)
**Employed**	
Yes	315 (84.9)
No	56 (15.1)
**Partnership**	
No	86 (23.2)
Yes	285 (76.8)
**people living in the household**	
1	47 (12.7)
2	133 (35.8)
3 ≥	191 (51.5)
**chronic physical illness**	
No	268 (72.2)
Yes	103 (27.8)
**mental disorder in the past**	
No	270 (72.8)
Yes	101 (27.2)
**days passed between participation in study and critical event**	132.42 (71.27)
**positive COVID test at some time after the critical event**	
no	253 (68.2)
Yes	118 (31.8)
**fear related to COVID-19** (range 1–7)	4.33 (2.22)
**self-efficacy** (range 1–7)	3.20 (1.85)
**risk perception** (range 1–7)	4.22 (2.19)
**knowledge about COVID-19** (range 1–7)	5.42(1.37)
**PHQ-4** (sum score)	8.42 (3.47)
**anticipated stigmatization**	
Yes	59 (15.9)
No	312 (84.1)
**internalized stigmatization**	
Yes	40 (10.8)
No	331 (89.2)
**disclosure concerns**	
Yes	116 (31.3)
No	255 (68.7)
**enacted stigmatization**	
Yes	75 (20.2)
No	296 (79.8)
**stigma practices**	
Yes	13 (3.5)
No	358 (96.5)

### 3.2 Latent class analysis

In our study sample, the 2-class solution (Log = -678.11, AIC = 1378.21, BIC = 1421.29, adjusted BIC = 1386.39, VLMR < 0.001, BLRT < 0.001, average latent class probabilities: 0.94, 0.92, entropy = 0.71) fitted the data better than the 3-class solution (Log = -672.23, AIC = 1378.45, BIC = 1445.03, adjusted BIC = 1391.09, VLMR = 0.06, BLRT = 0.12, average latent class probabilities: 0.95, 0.73, 0.97, and entropy = 0.83), based on AIC, BIC, adjusted BIC, average class probabilities, VLMR and BLRT value.

**Fig 1:**
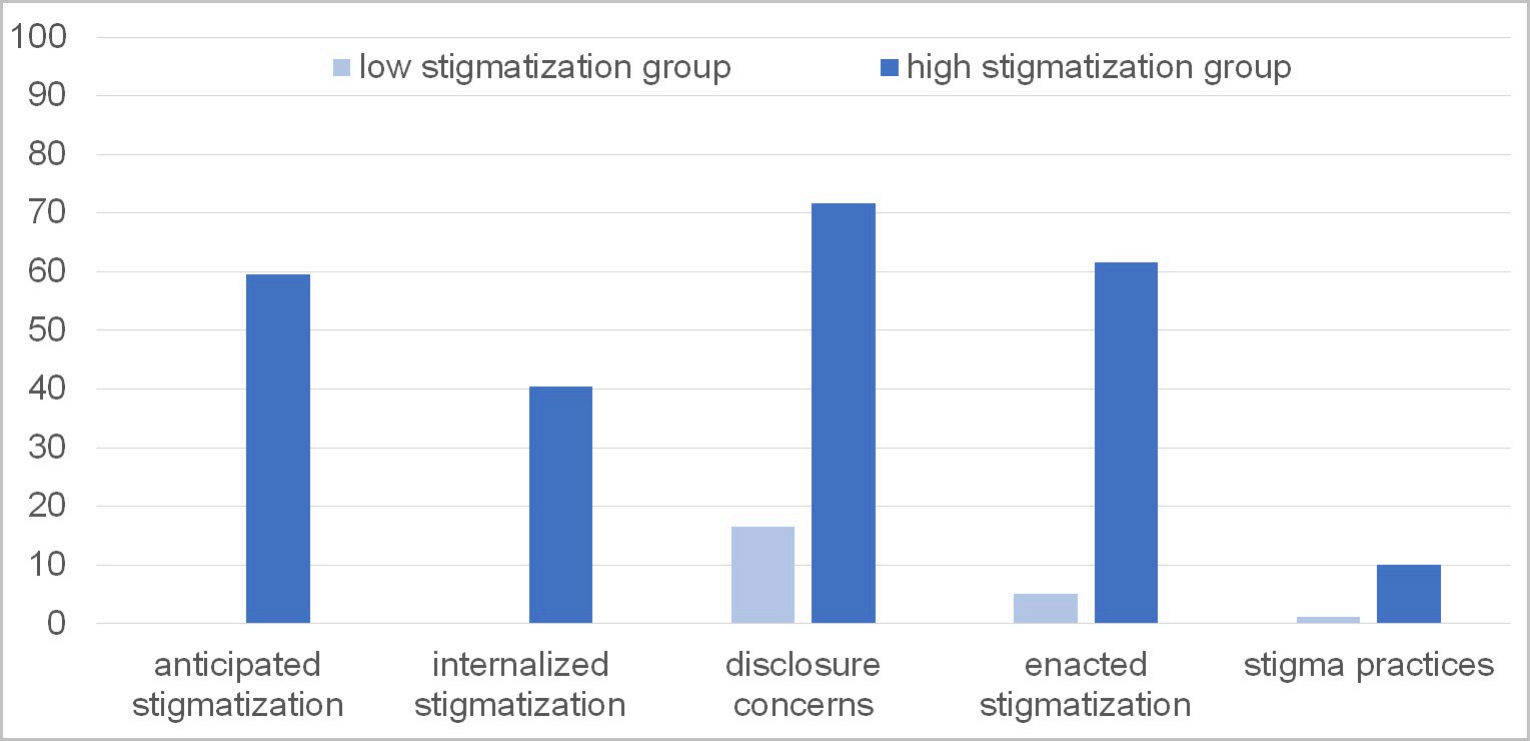
Stigmatization profiles. Relative endorsement rates (%) for anticipated stigma, internalized stigma, disclosure concerns, enacted stigma, and stigmatization practices in the two latent classes.

The following descriptions of latent class counts and proportions for the 2-class solution are based upon the most likely latent class membership. The first class was labeled “high stigmatization group” (26.69%) with moderate to high experienced stigmatization and low stigmatization practices, and the second class was labeled “low stigmatization group” (73,32%) with very low experienced stigmatization and stigmatization practices. The relative endorsement rates for anticipated stigma, internalized stigma, disclosure concerns, enacted stigma, and stigmatization practices of the participants in the two latent classes are shown in Fig 1. Descriptives for the two stigmatization classes are given in S1 Table.

### 3.3 Regression analyses

Wald statistics, odds ratios, confidence intervals for the odds ratios, and *p* values for the binary logistic regression analysis with stigmatization class as the dependent variable and demographic variables and psychological variables as independent variables are shown in Table 2A. The only demographic and psychological variables significantly related to stigmatization class were age, chronic physical illness, and fear related to COVID-19 within the first 24h after possible infection. Being younger (OR = 0.97; 95% CI: 0.950–0.995), not suffering from a chronic physical illness (OR = 0.55; 95% CI: 0.302–0.998), and having higher levels of fear related to COVID-19 within the first 24h after the critical event (OR = 1.170; 95% CI: 1.029–1.330) correlated with belonging to the „high stigmatization group“.

**Table 2:** Results of the regression analyses.

**a) logistic regression with stigmatization class as dependent variable**	**B**	**SE**	**Wald**	***p***	**OR**	**95% CI for OR**	
						**l bound**	**u. bound**
**Age**	**-0.028**	**0.012**	**5.503**	**0.019**	**0.973**	**0.950**	**0.995**
**Gender**	-0.313	0.316	0.980	0.322	0.731	0.393	1.359
**education**	0.531	0.306	3.012	0.083	1.701	0.934	3.100
**Employed**	-0.086	0.343	0.063	0.801	0.917	0.468	1.797
**Partnership**	-0.032	0.373	0.007	0.932	0.968	0.466	2.013
**1 person living in the household** **(reference: ≥ 3 people living in the household)**	-0.322	0.484	0.445	0.505	0.724	0.281	1.869
**2 people living in the household** **(reference: ≥ 3 people living in the household)**	-0.258	0.276	0.869	0.351	0.773	0.450	1.329
**chronic physical illness**	**-0.599**	**0.305**	**3.873**	**0.049**	**0.549**	**0.302**	**0.998**
**mental health disorder in the past**	-0.033	0.289	0.013	0.908	0.967	0.549	1.703
**days passed between participation in the study and critical event**	-0.003	0.002	2.469	0.116	0.997	0.994	1.001
**positive COVID test after the critical event**	-0.122	0.297	0.170	0.681	0.885	0.494	1.584
**fear related to COVID-19 (24h after having been at risk of exposition to COVID-19)**	**0.157**	**0.066**	**5.709**	**0.017**	**1.170**	**1.029**	**1.330**
**self-efficacy (24h after having been at risk of exposition to COVID-19)**	0.094	0.078	1.456	0.228	1.098	0.943	1.279
**risk perception ((24h after having been at risk of exposition to COVID-19)**	0.093	0.065	2.046	0.153	1.098	0.966	1.247
**knowledge about COVID-19 (24h after having been at risk of exposition to COVID-19)**	-0.085	0.098	0.760	0.383	0.918	0.758	1.112
**b) linear regression with PHQ-4 score as dependent variable**	**unstandardized** **coefficients**			**standardized coefficients**			
	**B**		**SE**	**β**		**t**	***p***
**Age**	-0.010		0.014	-0.035		-0.758	0.449
**Gender**	-0.181		0.380	-0.021		-0.476	0.635
**education**	0.100		0.345	0.013		0.289	0.773
**Employed**	-0.279		0.421	-0.029		-0.664	0.507
**Partnership**	-0.012		0.453	-0.002		-0.027	0.978
**1 person living in the household** **(reference: ≥ 3 people living in the household)**	0.084		0.583	0.008		0.144	0.885
**2 people living in the household** **(reference: ≥ 3 people living in the household)**	0.196		0.325	0.027		0.602	0.548
**chronic physical illness**	0.206		0.339	0.027		0.606	0.545
**mental health disorder in the past**	**0.844**		**0.339**	**0.108**		**2.492**	**0.013**
**days passed between participation in the study and critical event**	-0.002		0.002	-0.039		-0.887	0.376
**positive COVID test after the critical event**	**1.096**		**0.345**	**0.147**		**3.175**	**0.002**
**fear related to COVID-19 (24h after having been at risk of exposition to COVID-19)**	**0.497**		**0.075**	**0.318**		**6.640**	**< 0.001**
**self-efficacy (24h after having been at risk of exposition to COVID-19)**	**-0.276**		**0.090**	**-0.147**		**-3.063**	**0.002**
**risk of being infected (24h after having been at risk of exposition to COVID-19)**	**0.150**		**0.074**	**0.095**		**2.019**	**0.044**
**knowledge about COVID-19 (24h after having been at risk of exposition to COVID-19)**	**-0.322**		**0.117**	**-0.128**		**-2.765**	**0.006**
**stigmatization class (low = 0, high = 1)**	**2.022**		**0.336**	**0.258**		**6.015**	**< 0.001**

B, unstandardized regression coefficient; SE, standard error; Wald, Wald chi-square value; OR, odds ratio; l.bound, lower bound; u. bound, upper bound; β, standardized regression coefficient; t, t-value; *p*, 2 tailed *p*-value.

The model of the linear regression analysis with the PHQ-4 sum score as the dependent variable and demographic and psychological variables (including stigmatization class) as independent variables was significant (F = 14.22, p < 0.001) with 36% explained variance (adj R^2^ = 0.36). Higher scores on the depression scale were significantly related to a mental health disorder in the past (β = 0.11, p = 0.013), positive COVID-test after the critical event (β = 0.15, p = 0.002), fear related to COVID-19 within the first 24 h after the critical event (β = 0.32, p<0.001), self-efficacy within the 24 h after the critical event (β = -0.15, p = 0.002), perceived risk of having been infected with COVID-19 within the 24 first h after the critical event (β = 0.10, p = 0.044), perceived amount of knowledge about COVID-19 during the first 24h after the event (β = -0.13, p = 0.006) and stigmatization class (β = 0.26, p<0.001). Unstandardized and standardized coefficients, standard errors, t-values, *p*-values, and effect sizes are shown in Table 2B.

## 4. Discussion

To our knowledge, this is one of the first studies providing extensive stigmatization patterns for COVID-19-related stigmatization in a German population at high risk of infection using latent class analysis. The relatively low rates of stigmatization in our study sample align with previous studies on COVID-19-related stigmatization in the German population [36,37]. In one study, stigmatizing attitudes towards people suffering from COVID-19 were investigated using two subscales „support for discrimination”and „blame”in 157 German participants in March 2020 [36]. The authors reported low to moderate amounts of stigmatization (${\bar{x}}_{\mathrm{support}\;\mathrm{for}\;\mathrm{discrimination}}$ = 2.5 and ${\bar{x}}_{\mathrm{blame}}$ = 1.4; scales range = 1–5) in the general population. Other authors investigated the hypothesis that stigmatization of Chinese and Asian-looking people increases in the general population of Germany with rising numbers of people infected with SARS-CoV-2 [37]. They found no evidence of an increase in the stigmatization of Chinese and Asian-looking people on interpersonal and societal levels. All in all, the results of the German studies show smaller amounts of stigmatization than one might have expected based on the estimated pooled prevalence of 27% (95% CI: 18–36) for COVID-19-related stigmatization in high-income countries reported in a recent meta-analysis on COVID-19-related stigmatization [6]. One possible explanation might be that most of the participants in the German studies had high levels of education and might therefore have had more accurate knowledge about infectious diseases and been less prone to believe in misinformation about COVID-19 contagion, which has been shown to be one of the main drivers of stigmatization [6].

### 4.1 Stigmatization profiles

We found two stigmatization profiles among our study sample, which we labeled “high stigmatization group” and “low stigmatization group”. The “high stigmatization group” displayed moderate anticipated stigma, low internalized stigma, moderate enacted stigma, high disclosure concerns, and very low stigma practices, while the “low stigmatization group” showed no anticipated stigma, no internalized stigma, and very low rates of enacted stigmatization, disclosure concerns, and stigma practices. Interestingly, in the “high stigmatization group”, not all participants stated having felt internalized stigmatization, which aligns with a previous study on stigmatization profiles in people at risk of infection [23]. These authors investigated perceived courtesy stigma (concern with public attitudes against people of Hubei) and affiliate stigma (internalized stigmatization) in people of Hubei who were considered people at risk of contagion due to their geographic linkage to COVID-19. They found three latent classes of stigmatization groups, which they labeled “Denier” (35.98%), “Confused moderate” (48.13%), and “Perceiver” (15.89%). Similarly to our results, their „Perceiver”class was the smallest class, and although participants belonging to this class reported the highest stigma, they did not agree with the item “Because the COVID-19 outbreak took place in Wuhan/Hubei, I feel shamed and self-blame” tapping into internalized stigmatization. The authors suggested as a possible explanation that participants belonging to the „Perceiver”class might already have realized that the stigmatizing behaviors of others are inappropriate. Realizing that stigmatizing behavior is inappropriate, along with possible social desirability effects, might also be a reason why stigmatization practices were rather low in our “high stigmatization group”. The only demographic and psychological variables significantly related to belonging to the “high stigmatizing group” in our study sample were younger age, not suffering from a chronic physical illness, and higher fear related to COVID-19. Younger age has been related in several studies to higher amounts of COVID-19-related stigmatization and, in some studies, to lower levels of stigmatization [6,8,38]. While we found that people suffering from a chronic illness reported rather less stigmatization, other authors found the opposite [8]. Fear of COVID-19 has been related repeatedly to higher levels of stigmatization in other studies [8,39–41]. From an evolutionary perspective, this relationship might be explained by the fact that humans developed a disease-avoidance mechanism that causes people to be selective in their social interactions to protect their physical integrity [12,42].

### 4.2 Relationship between stigmatization profiles and psychological distress

The second aim of our study was to investigate the relationship between stigmatization and psychological distress while taking into account other possible demographic and psychological risk factors. In our study sample, belonging to the “high stigmatization group” correlated with higher psychological distress, which aligns with previous studies [9,43,44]. Stigmatization and discrimination experiences can be considered stressors leading to reduced mental health [45]. However, as our results can only be interpreted at a correlational level, an alternative explanation might be that psychological distress produces cognitive biases influencing the information processing of social acceptance cues which might lead to a higher perception of experienced stigmatization [46,47].

### 4.3 Other risk factors for psychological distress

As far as the other investigated positive and negative risk factors for psychological distress are concerned, our results are in line with previous research showing a positive correlation with a mental health disorder in the past [9,48], tested positively for COVID-19 [49], fear related to COVID-19 [8,39,41,48], perceived risk of being infected [9,44,50] and a negative correlation with perceived self-efficacy [9,51], and subjective level of knowledge about COVID-19 [9].

However, our study design did not capture all possible confounding risk factors for COVID-19-related psychological distress. Other authors showed, for instance, that fear of personal death [52], intolerance of uncertainty [53], emotion regulation strategies [53,54], maladaptive coping strategies [55], and personality traits [52,56] also constitute personal risk factors for reduced mental health during COVID-19.

In addition to these personal risk factors associated with the health threat posed by COVID-19, there are also risk factors resulting from the large changes in everyday life due to the imposed lockdowns, quarantine, isolation and public health measures [2,5,57]. While people were forced to stay at home and keep physical distance, their activities shifted from social activities to more sedentary indoor activities like, for instance, problematic social media use [58]. Problematic social media use has been shown to enhance fear of COVID-19 as well as misunderstanding of COVID-19, both leading to increased psychological distress [58]. Furthermore, the sharp reduction in social activities created (especially for young adults) discrepancies between actual and desired levels of social interaction, leading to increased feelings of loneliness which have been associated with psychological distress [59,60]. Other pandemic-related changes that have been shown to influence mental health are, for instance, changes in the mode of working, job loss, and associated financial losses [2,60–62].

## 5. Limitations

Several limitations in this study should be acknowledged. Our number of participants was slightly smaller than the conservatively calculated required sample size of 384 participants. Furthermore, the recruitment method of the study sample may have led to a selection bias and our study sample might thus not be representative of the German population. As data acquisition took place during the COVID-19 pandemic, we conducted an online survey via convenience sampling, which is feasible and safe considering the transmission of the coronavirus. As a result, people with limited access to the internet (e.g., people with low income, elderly people) were less likely to participate in our study, while women, who have been shown to be more interested in online surveys than men [63], were more likely to participate in our study. Although our study sample is rather homogeneous (mainly female, middle-aged, well-educated, and without migration background), we still observed substantial heterogeneity in our data.

Furthermore, as our study relies on self-reported data, the low reported stigmatization practices might partly be due to social desirability effects. Another limitation of this study is that we used stigmatization items adapted from well-validated stigma scales for HIV and chronic diseases since no standardized stigmatization scale for COVID-19 was available at the time of the recruitment. Although our scales were reliable according to the respective Cronbach’s alphas and face validity, intelligibility and German translation (of the anticipated stigmatization items and stigmatization practices items) had been checked by all research group members not involved in this study, the psychometric properties of our stigmatization questionnaire remain uncertain as it has not been thoroughly validated within a pilot study. Additionally, other possible positive and negative risk factors for psychological distress were assessed in the current study with single items which might not fully cover the respective construct. Especially fear of COVID-19 could have been more accurately assessed using the Fear of COVID-19 Scale [64,65]. However, it should be noted that these constructs were not the main focus of this study and were principally included as control variables to ensure that potential effects of stigmatization on psychological distress are not due to confounding variables. Residual confounding bias is nevertheless still possible since there still exist relevant constructs that were not assessed in this study.

## 6. Conclusions

We investigated stigmatization profiles for COVID-19-related stigmatization as well as the relationship between stigmatization profiles and psychological distress in a German population at high risk for COVID-19 infection while taking into account possible demographic and psychological risk factors. The strength of our study is that it adds to the body of knowledge about stigmatization in high-income countries. Furthermore, it is one of the first studies using LCA to provide a detailed profile of COVID-19-related stigmatization. Our “high stigmatization group” displayed moderate anticipated stigma, low internalized stigma, moderate enacted stigma, high disclosure concerns, and very low stigma practices. Especially, high disclosure concerns might involve a certain risk that the individuals in question do not use health services for diagnostics or treatment to avoid stigmatization and discrimination. This can have detrimental effects on their health and hampers public health efforts to check the spread of the disease. Further anti-stigmatization campaigns, even in countries where more obvious forms of stigmatization are less present, are therefore needed to reduce the disclosure concerns of people at high risk for infection and to eliminate stigmatization as a potential risk factor for psychological distress. Additionally, policymakers should be trained in using evidence-based health communications in official speeches to destigmatize COVID-19 further. Additional longitudinal research investigating stigmatization profiles with standardized questionnaires in large, representative study samples is needed to corroborate the findings of the current study.

## Supporting information

S1 Table Descriptive data for the two stigmatization classes. (DOCX)

S1 File Minimal underlying dataset. (XLSX)
