# Supplementary material for: Stigmatization profiles and psychological distress in people at high risk of infection with COVID-19 –A study conducted in Germany from March to August 2021
Source: PLoS One. 2023 May 18;18(5):e0285788. doi: 10.1371/journal.pone.0285788 (PMC10194892; doi:10.1371/journal.pone.0285788)
Supplement: S1 Table — (DOCX) [file pone.0285788.s001.docx]

|  | **high stigmatization group (n=99)**  mean (± SD) / n (%) | **low stigmatization group (n=272)**  mean (± SD) / n (%) |
| --- | --- | --- |
| **age** (range 18-73 years) | 39.56 (10.07) | 43.44 (12.08) |
| **gender** |  |  |
| male | 22 (22.2) | 53 (19.5) |
| female | 77 (77.8) | 219 (80.5) |
| **nationality** |  |  |
| German | 97 (98.0) | 272 (100) |
| other | 2(2.0) | 0 (0) |
| **education level** |  |  |
| < 12 years | 20 (20.2) | 75 (27.6) |
| > 12 years | 79 (79.8) | 197 (72.4) |
| **employed** |  |  |
| yes | 81 (81.8) | 234 (86.0) |
| no | 18 (18.2) | 38 (14.0) |
| **partnership** |  |  |
| no | 23 (23.2) | 63 (23.2) |
| yes | 76 (76.8) | 209 (76.8) |
| **people living in the household** |  |  |
| 1 | 12 (12.1) | 35 (12.9) |
| 2 | 30 (30.3) | 103 (37.9) |
| 3 ≥ | 57 (57.6) | 134 (49.3) |
| **chronic physical illness** |  |  |
| no | 79 (79.8) | 83 (30.5) |
| yes | 20 (20.2) | 189 (69.5) |
| **mental disorder in the past** |  |  |
| no | 72 (72.7) | 198 (72.8) |
| yes | 27 (27.3) | 74 (27.2) |
| **days passed between participation in study and critical event** | 123.33(70.79) | 135.72(71.28) |
| **positive COVID test at some time after the critical event** |  |  |
| no | 69 (69.7) | 184 (67.6) |
| yes | 30 (30.3) | 88 (32.4) |
| **fear related to COVID-19** (range 1-7) | 4.64 (2.16) | 4.22(2.23) |
| **self-efficacy** (range 1-7) | 3.17(2.07) | 3.21(1.76) |
| **risk perception** (range 1-7) | 4.38 (2.22) | 4.17 (2.18) |
| **knowledge about COVID-19** (range 1-7) | 5.32(1.43) | 5.46 (1.36) |
| **PHQ-4** (sum score) | 10.14(3.58) | 7.79 (3.22) |
| **anticipated stigmatization** |  |  |
| yes | 59 (59.6) | 0 (0) |
| no | 40 (40.4) | 272 (100) |
| **internalized stigmatization** |  |  |
| yes | 40 (40.4) | 0 (0) |
| no | 59 (59.6) | 272 (100) |
| **disclosure concerns** |  |  |
| yes | 71 (71.7) | 45 (16.5) |
| no | 28 (28.3) | 227 (83.5) |
| **enacted stigmatization** |  |  |
| yes | 61 (61.6) | 14 (5.1) |
| no | 38 (38.4) | 258 (94.9) |
| **stigmatization practices** |  |  |
| yes | 10 (10.1) | 3 (1.1) |
| no | 89 (89.9) | 269 (98.9) |

**S1 Table. Descriptives for the two stigmatization classes.**
